# Deep learning-based classification of anti-personnel mines and sub-gram metal content in mineralized soil (DL-MMD)

**DOI:** 10.1038/s41598-024-60592-8

**Published:** 2024-05-11

**Authors:** Shahab Faiz Minhas, Maqsood Hussain Shah, Talal Khaliq

**Affiliations:** 1 CESAT, Islamabad, Pakistan; 2 School of electronics and computing, Dublin City University, Dublin, Ireland

**Keywords:** Signal processing, CNN, Artificial intelligence, Neural networks, Pulse induction, Electrical and electronic engineering, Software

## Abstract

De-mining operations are of critical importance for humanitarian efforts and safety in conflict-affected regions. In this paper, we address the challenge of enhancing the accuracy and efficiency of mine detection systems. We present an innovative Deep Learning architecture tailored for pulse induction-based Metallic Mine Detectors (MMD), so called DL-MMD. Our methodology leverages deep neural networks to distinguish amongst nine distinct materials with an exceptional validation accuracy of 93.5%. This high level of precision enables us not only to differentiate between anti-personnel mines, without metal plates but also to detect minuscule 0.2-g vertical paper pins in both mineralized soil and non-mineralized environments. Moreover, through comparative analysis, we demonstrate a substantial 3% and 7% improvement (approx.) in accuracy performance compared to the traditional K-Nearest Neighbors and Support Vector Machine classifiers, respectively. The fusion of deep neural networks with the pulse induction-based MMD not only presents a cost-effective solution but also significantly expedites decision-making processes in de-mining operations, ultimately contributing to improved safety and effectiveness in these critical endeavors.

## Introduction

Metal-based mine detectors have been extensively employed in humanitarian and military de-mining operations for the past seventy years. However, as technology advances, new challenges and difficulties arise. Notably, the metal content in mines, particularly anti-personnel mines and integrated explosive devices (IEDs), is decreasing, while the quantity of trash and miscellaneous metal per square meter is increasing due to human activities and conflicts. According to the UN de-mining operation report^1^, for every mine found, there are about a thousand small pieces of scrap metal detected.

In the past decade, the notable advancement in demining endeavor has been the introduction of Ground Penetrating Radar (GPR). However, for practical mine detection purposes, GPR still requires the support of a metal detector. This combination of GPR and a metal detector, known as a hybrid technology^2^, is currently utilized by advanced military forces worldwide. In this case, the decision based on the detection can be independent of each other using separate signal processing techniques or can be a combination of (both) using data fusion algorithm as shown in Ref^3^. Some widely used GPR-based mine detectors include the Vallon Minehound VMR3 and CEIA ALIS-RT etc.^4,5^. Nonetheless, this technology is extremely expensive when compared to traditional metal detectors, as it costs eight to ten times more approximately (Ref^4^, quote can be obtained via contact) and still relies on an integrated metal detector sensor.

Over the years, there have been significant advancements in the search head assembly of metallic mine detectors, including the use of multi-strand coils^6^ to enhance sensitivity and the implementation of various factors to reduce noise. However, the true potential of metallic mine detectors (MMDs) remains largely untapped, leaving ample room for improvement, particularly in the field of signal processing. Given the progress in machine learning and robust artificial intelligence techniques, it is a logical progression to explore the application of these techniques in MMDs. Recent mine detectors have already incorporated machine learning techniques with a limited focus, such as automatic soil compensation and the differentiation of nonferrous and ferrous materials^7^. A similar concept from GPR (2D image) is utilized using MMDs only^8^, with a machine learning based classification of mines & metals. It is limited in terms of classification accuracy in soil and is also limited in terms of practical utility (explained later).

In the study discussed in Ref^9^, the focus is on binary classification—determining whether an object is a mine or not with using spatial measurement diversity. The results indicate best performance of a 50% probability of detection (& identification) of mines with minimal false alarm rate (of less than 5%). However, this rate increase substantially (above 30%) with the increase in detection probability especially for mines with low metal content (like APM), which render it impractical. Another approach, detailed in Ref^10^, involves a custom architecture with two receiver coils and one transmitter coil, which utilizes broadband frequencies (ranging from 60 Hz to 15.8 kHz) and employs an inversion procedure to estimate the magnetic properties of metal targets. Unlike typical MMDs, this method is distinct in its complexity, as instead of moving the electromagnetic interference (EMI) sensor over the target, the target itself is moved over the sensor. It is neither feasible to practically move such large rectangular EMI sensor by a user in a field environment over a target nor authors have shown/discussed its efficacy against any buried target.

In order to circumvent the limitations such as limited accuracy, high complexity, limited practical utility, in this research, we utilize a pulsed induction (PI) metal detector. This type of technique has advantages in various scenarios where other non-PI based detectors (e.g. CW) would face challenges^11^, particularly in environments with highly conductive materials in the soil or surroundings. Additionally, PI-based systems have the ability to detect metal at greater depths compared to other systems. In the following section, we will provide a brief explanation of the operation of a PI detector. However, it is important to note that in mine detection algorithms, a significant portion of the received signal curve information (sample by sample) is not extracted, as the focus is primarily on integration (of the curve samples)^12^ and filtering to ensure reliability and user safety. Our research, on the other hand, centers on this received signal curve and will be presented in the subsequent sections.

The main contributions of this paper are highlighted in the following:

1. Design of data acquisition front end to ensure amplified signal with low noise and enhanced detection capability.
2. Development of a post-detection classification algorithm based on a novel deep neural networks (DNN) architecture.
3. Creation of a comprehensive dataset through practical scenarios in a laboratory environment to facilitate algorithm development and evaluation.
4. Application of the proposed algorithm to accurately classify detected targets as either anti-personnel mines (APMs) or non-APMs in both mineralized soil and non-mineralized environments (air or sand).
5. Achievement of a high validation accuracy of 93.5% for the proposed novel algorithm on the dataset, showcasing its effectiveness in mine classification.

Rest of the paper is organized such that, the current systems’ limitations and problems are provided in Sect. “Pulse induction MMD”, followed by the discussion of data acquisition and the motivation of proposed algorithm in Sect. “Data acquisition”. The proposed AI-based classification algorithm is presented in Sect. “Proposed AI model”. Section “Simulations, results & discussions” will cover simulations and results, and finally, Sect. “Conclusion” will present the conclusion. To the best of our knowledge the work proposed in this paper has not been tackled in this manner in literature. We provide the open-source code and datasets (MinhasSF/MMD_AI (github.com)) for further improvements in a collaborative manner.

## Pulse induction MMD

The PI detector used in this research has a single sensor coil, comprising of multi-strands wire. The advantage of using single coil for transmitter & receiver (Tx/Rx) will ensure a same channel response for transmitted and received signals. The PI metal detector sends powerful, short bursts (pulses) of current through a coil of (multi-strands) wire which will magnetize the sensor coil at the rate of 1150 μs (approx.) for positive polarity pulses and similar is the case for negative polarity pulses. The reason for using the bipolar pulses is to reduce the risk of triggering magnetically-activated mines and booby-traps^13^. The pulse time of single transmission is about 45–65 μs to charge (magnetize) sensor coil and hence produce magnetic flux to charge magnetic material (targets) around it. When the pulse ends, charged coil demagnetizes and magnetic flux around it collapses producing flyback voltage of few hundred volts (i.e. sharp electrical spike) at sensor coil. Magnetic flux collapse induces eddy current in target material which oppose demagnetization of sensor coil. This opposed demagnetizing effect by a certain decay rate is a characteristic of metal content in the target^7^.

In case of mineralized soil, the field has magnetization properties similar to metal target. This decay rate is deterministic and can be referred to as its signature. The strength of this magnetized soil on the sensor coil can be larger than coming from a target with small metal content especially in an APM which can be less than 0.3 g or even 0.2 g. As per Geneva convention of amended protocol II^14^ on certain conventional weapons, an APM must have 8 g of detection metal plate but is not considered in this research. To detect the target APM buried in mineralized soil, machine learning tools are used by different mine detectors for example Vallon VMH3, VMH4^4^ and others that generally learn this predictable signal and then removes it from the received signal and thus the remaining signal is due to the target. The machine learning employed is on spot learning that does the mineralized soil compensation (automatically) only. The machine learning employed is limited in scope and is typically without any hidden layers, this compensation will be referred to as machine learning based compensation.

To ensure reliability, the compensated signal (less mineralized signal) is integrated and after passing through post-processing algorithms, it is finally fed to a comparative threshold-based alarm generator. It is indeed a very reliable system (with negligible false negative), as the target metal content shape, size, type, depth and orientation that are fundamental to classification are put aside. Any information present within the compensated signal is not considered, only strength of integrated signal will define the target through sound (louder the sound the bigger is the target and vice versa). With this, the decision making of calling it a target of concern comes down to the experience and instincts of a trained user. Prior to integration, it is still crucial to extract additional information for classification to aid in the decision-making process.

At this point, it is relevant to discuss a case that uses post-integrated dataset for machine learning based classification in Ref^8^. For this a grid of [11 × 10] data points is created and a robotic arm is used to sweep the area of size 60 cm × 50 cm. The authors use a deep convolutional neural network composed of several non-linear transformations. Instead of extracting information from the received signal at a particular location within the grid, the whole received signal is integrated into one data point or one sample per location which leads to a limited performance. In this research the focus is on the received signal curve and the information present in it, will be discussed in the following.

## Data acquisition

**Figure 1:**
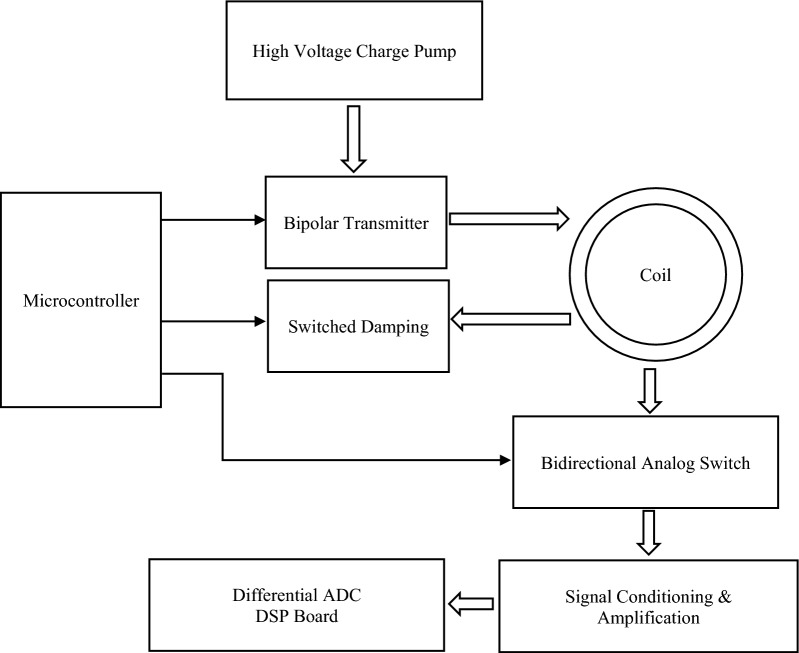
The block diagram of pulse induction MMD showing transmitter (Tx), receiver (Rx) and the sensor coil.

In order to understand the received signal, it is essential to first briefly describe the working of transmitter and receiver of PI MMD used in this research (block diagram shown in Fig. 1). Transmitter (Tx) works on pulse induction principle with high voltage charge pump and switched coil damping. Usually metal detectors Tx described as total loss system flyback voltage (energy produced due to magnetic field collapse) is damped through ohmic resistance causing loss of energy (see pp 143–145 of Ref^15^). In current system (Earle Model is followed^16^) Tx flyback energy is stored in capacitor(s). Hence, energy loss from flyback energy is conserved for coil charge in subsequent cycle where energy is transferred from charged capacitor(s) to coil in capacitor/inductor resonance with frequency of ½ $\uppi$ and then continues with normal low voltage charging to sustain the current (and magnetic field) across the coil. This method generates approximately constant current and constant magnetic field across coil during charging as opposed to negative exponential current in total loss system. (for details see Figs. 3 and 4 in Ref^16^).

At the end of charging cycle, coil current (magnetic flux) collapses creating voltage flyback which charges capacitor(s) for 2us to repeat the cycle (self). After 2us, rest of the energy is damped through ohmic resistance in switched damping circuit, to get shortest possible time constant that a target can have to be detected. Receiver blocking circuit is composed of bidirectional analog switch which isolates receiver (Rx) circuit with Tx circuit and also provides some resistance to high voltage signal.

**Figure 2:**
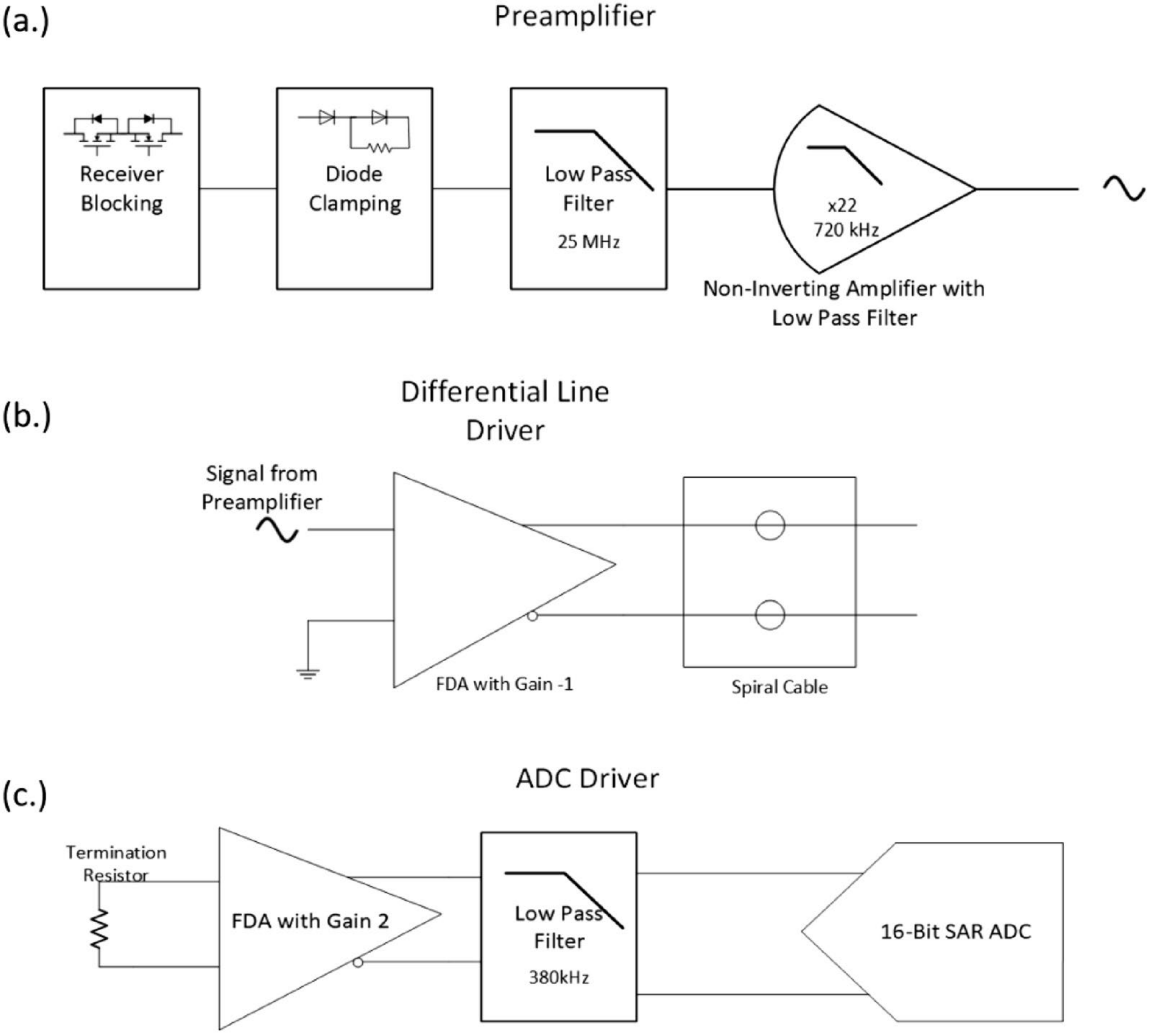
Signal chain of data acquisition system.

The receiver (Rx) circuit (for detailed working see pp 75–77 Ref^15^), is composed of voltage clipping, conditioning and amplification. Voltage needs to be clipped to work in connivance with sensitive electronics along with signal conditioning which involves low pass filters. The received signal undergoes clamping and reversal as a result of the internal circuitry of the receiver switch and the positioning of diodes. For completeness, the received signal chain, depicted in Fig. 2, will be briefly discussed here.

**Figure 3:**
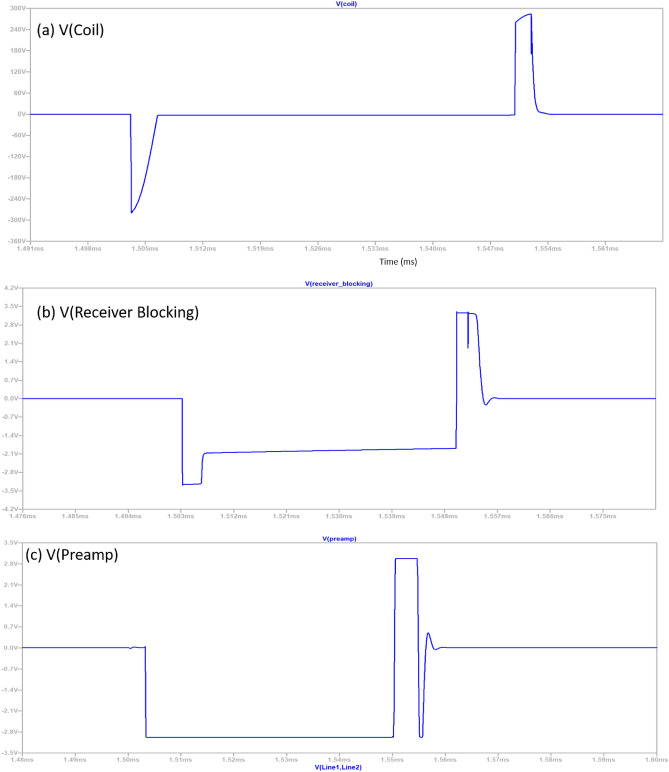

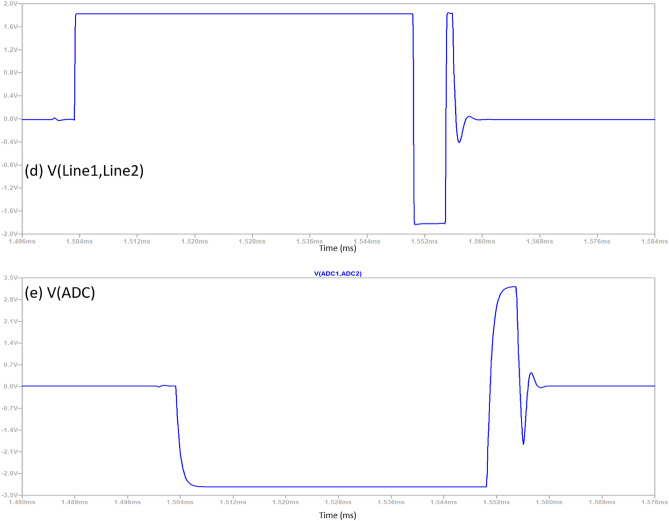
Stage wise pictorial view of received signal of negative pulse at different stages of signal chain of data acquisition.

The signal chain of the data acquisition system is depicted in Fig. 2, and a pictorial view of the signal at each stage is shown in Fig. 3. The system is divided into three parts: (a) preamplifier, (b) differential line driver, and (c) ADC driver. Before preamplifier receiver blocking will be briefly discussed, as the name implies, safeguards sensitive components in data acquisition systems from flyback voltage. It accomplishes this by establishing a resistive pathway alongside shunt clamping diodes, ensuring that the signal remains within the safe operating voltage range. In preamplifier stage, the incoming signal undergoes signal conditioning, which includes passing through a two-pole 25 MHz low-pass filter. After this initial step, it proceeds through a non-inverting amplifier with 22 times amplification, followed by another low-pass filter with a cut-off frequency of 720 kHz.

The incoming signal is converted to a differential signal using a Fully Differential Amplifier (FDA) with a gain of − 1 while rejecting common-mode noise. This FDA functions as a Differential Signalling Line Driver similar to Low Voltage Differential Signalling (LVDS), ensuring that the signal is transmitted over a spiral cable wire with matched impedance accepted by termination resistor at the end spiral cable.

Hereafter, Fully Differential Amplifier (FDA) accepts a differential input and produces a differential output. It serves as the driving stage for a 16-bit Successive Approximation Register Analog-to-Digital Converter (SAR ADC) working at 2 Mega samples/s. Additionally, the signal path includes a single-pole RC filter of bandwidth of 380 kHz for charge transient created by ADC during sampling process. The resultant signal can be sampled using an ADC once it has settled to the ground with small “kink”.

**Figure 4:**
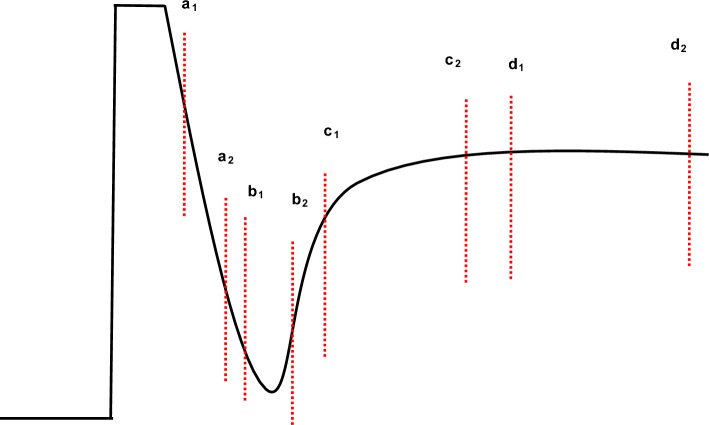
Received signal picked up by the sensor coil and has passed through pre-processing.

The received signal shown in Fig. 4 is ideal as it does not include any noise and interference and also the shape of curve may vary as it is based on architecture of search-head of MMD, in our case coil and Tx/Rx assembly. The sensitive area of the curve starts (just after the “kink”) from left to right and is being marked in sections (i.e. from a_1_ to a_2_, b_1_ to b_2_, c_1_ to c_2_ & d_1_ to d_2_), where very low metal contents are mostly present in the initial sections. The decay rate is being calculated using signal processing techniques on different sections. The sections width, the start, the end, overlapping and redundancy etc., are few factors that determine the robustness of compensation. It can be done manually or through machine learning (mostly on spot learning); however, the scope of this paper is not to discuss the soil compensation or to remove mineralization effect.

The purpose of this paper is to go beyond compensation and to do mine/metal classification by fully utilizing the received signal curve in different environments *i.e.* mineralized and non-mineralized environments (air & sand). The reason for applying AI technique will also become evident later on in next section.

To ensure completeness, we will now address the critical aspect of achieving pinpoint accuracy. Typically, a metallic mine detector operates in two modes: normal and mineralized (on-spot learning). Once an object buried underground is detected and identified as the target of interest by a trained operator using these modes, the subsequent task is to precisely locate the target's position. This precision is achieved by sweeping the search-head, oriented parallel to the ground, of the MMD multiple times over the target from various angles. The strongest signal is typically detected when the target is positioned at the center of the coil or search-head, ensuring an accurate pinpoint location. Subsequently, a classification algorithm (which will be discussed in the following section) becomes essential to classify the detected target, in our case, an APM mine. For pinpointing, normal mode is recommended in this research i.e. without machine learning based soil compensation since it is replaced by a novel DL-MMD classifier.

## Proposed AI model

**Figure 5:**
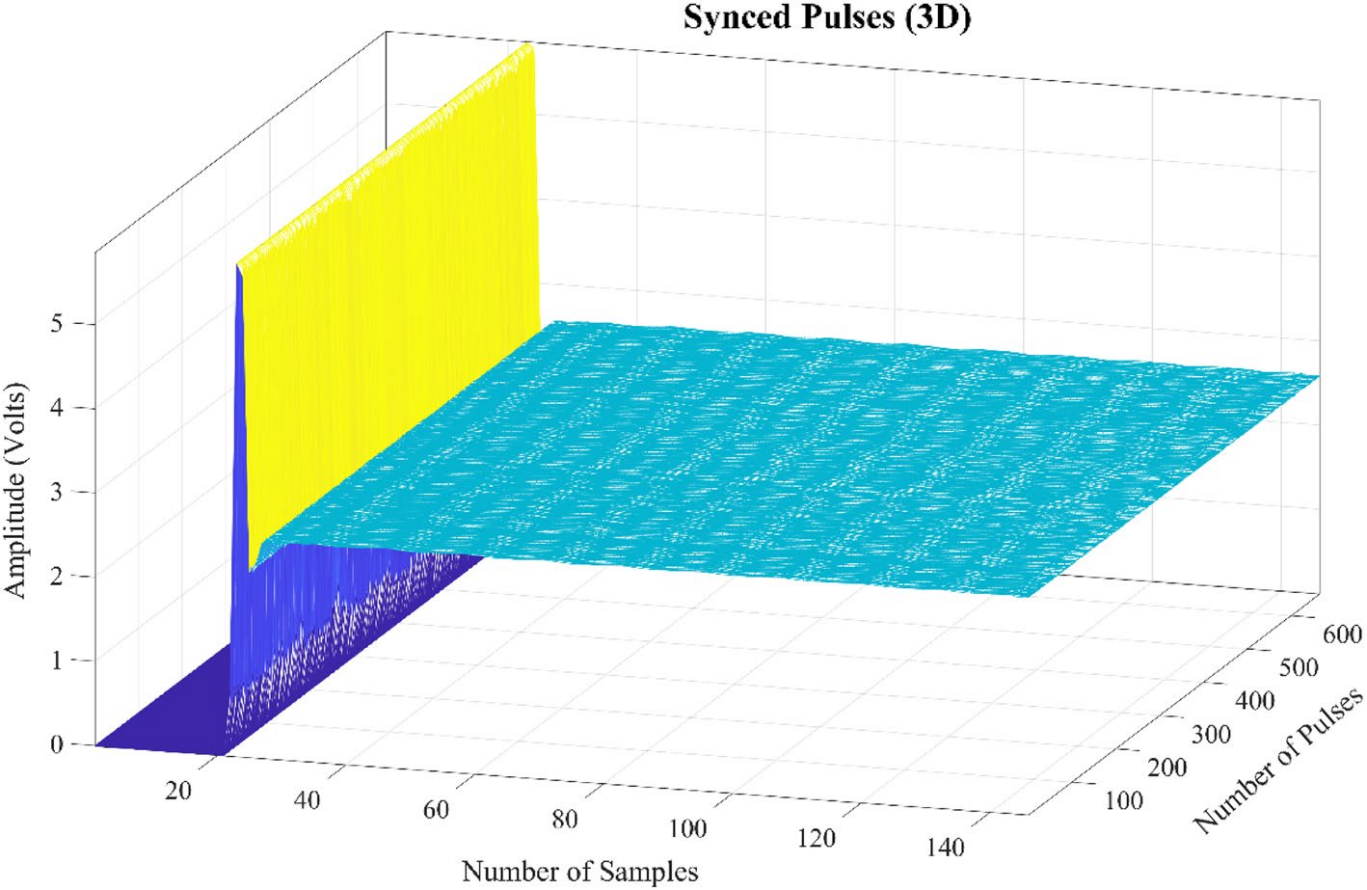
The 3D synced received signal of air, the number of pulses is equal to 665 and number of samples per pulse is equal to 150. It is important to point out that only a limited number of samples are shown of a negative pulse at sampling rate of 2 MHz. Similar is the case for positive pulse.

Prior to delving into the algorithm architecture, we will first focus on the number of classes that are to be classified and the dataset. Typically, in soil compensation we have two classes one is air and the other is mineralized soil. Air means that either the sensor coil has no mineralized soil in its proximity (just air) or the soil in proximity is not mineralized. Mostly the first option is considered otherwise there can be a slight bias in decay rate due minute mineral content within non-mineralized soil. However, in this research both are considered and for the later one sand is used. The default shape of the curve in which nothing is in proximity of the sensor coil, it will be called air (class A). The other eight classes are when sensor coil is exposed to or in proximity of mineralized soil (class B), sand (class C), APM (class D), vertical paper pins (class E), APM in presence of mineralized soil (class F), APM in presence of sand (class G), vertical paper pins in presence of mineralized soil (class H) and vertical paper pins in presence of sand (class I). The class A dataset is shown by a matrix ${{\varvec{P}}}_{{\varvec{A}}}$ as below:

1 $${\varvec{P}}_{{\varvec{A}}} = \left[ {\begin{array}{*{20}c} {P\left( {f{1}} \right)} \\ {P\left( {f{2}} \right)} \\ {P\left( {f{3}} \right)} \\ \vdots \\ {P\left( {f{\text{N}}} \right)} \\ \end{array} } \right] = \left[ {\begin{array}{*{20}c} {p\left( {f{1},n{1}} \right)} & {p\left( {f{1},n{2}} \right)} & \cdots & {p\left( {f{1},n{\text{M}}} \right)} \\ {p\left( {f{2},n{1}} \right)} & { p\left( {f{2},n{2}} \right)} & \cdots & { p\left( {f{2},n{\text{M}}} \right)} \\ {p\left( {f{3},n{1}} \right)} & {p\left( {f{3},n{2}} \right)} & \cdots & {p\left( {f{3},n{\text{M}}} \right)} \\ \vdots & \vdots & \ddots & \vdots \\ {p\left( {f{\text{N}},n{1}} \right)} & {p\left( {f{\text{N}},n{2}} \right)} & \cdots & { p\left( {f{\text{N}},n{\text{M}}} \right)} \\ \end{array} } \right]$$

where $P\left(f{1}\right)$ is a vector containing the concatenated received signal coming from the positive and negative transmitted pulses. The total number of pulses per class are N and the total number of samples per pulse (inclusive of both positive & negative) are given by M. Figure 5 shows the 3D image of the digitally synced received signal that is used to populate the dataset ${{\varvec{P}}}_{{\varvec{A}}}$, where x-axis shows the number of samples in a pulse, *y*-axis shows the amplitude in volts and *z*-axis shows the number of pulses. It can be observed from the figure that there is a slight variation from pulse to pulse that can be due to either thermal noise or due to any external weak interfering signal. The number of synced pulses shown are 665 of the negative pulses only (for simplicity) with time duration of 75 μs per pulse at a sampling rate of 2 MHz. However, the dataset ${{\varvec{P}}}_{{\varvec{A}}}$ contains the data from both positive and negative pulses, obtained just after the kink i.e. 122 samples (61 μs) per pulse. Similar matrices ${{\varvec{P}}}_{{\varvec{B}}}$ ,${{\varvec{P}}}_{{\varvec{C}}}$ , ${{\varvec{P}}}_{{\varvec{D}}}$, ${{\varvec{P}}}_{{\varvec{E}}}$, ${{\varvec{P}}}_{{\varvec{F}}}$**,**${{\varvec{P}}}_{{\varvec{G}}}$, ${{\varvec{P}}}_{{\varvec{H}}}$ and ${{\varvec{P}}}_{{\varvec{I}}}$ are for other eight classes. For each class, there will be a one-hot encoded vector representing the label, which is represented by a matrix with dimensions [N × 9].

In the last section, we have discussed the soil compensation robustness briefly in which two areas of the received signal curve processing needs to be pointed out here i.e. overlapping of sections and redundancy of channels. The overlapping and redundancy are to ensure that the signature of mineralized soil does not get mixed with the signature of other materials that might have close decay rate. So, a multi-channel approach is already present in soil compensation mode. In addition to this, the presence of positive and negative pulses of PI system, increase the numbers of channels.

**Figure 6:**
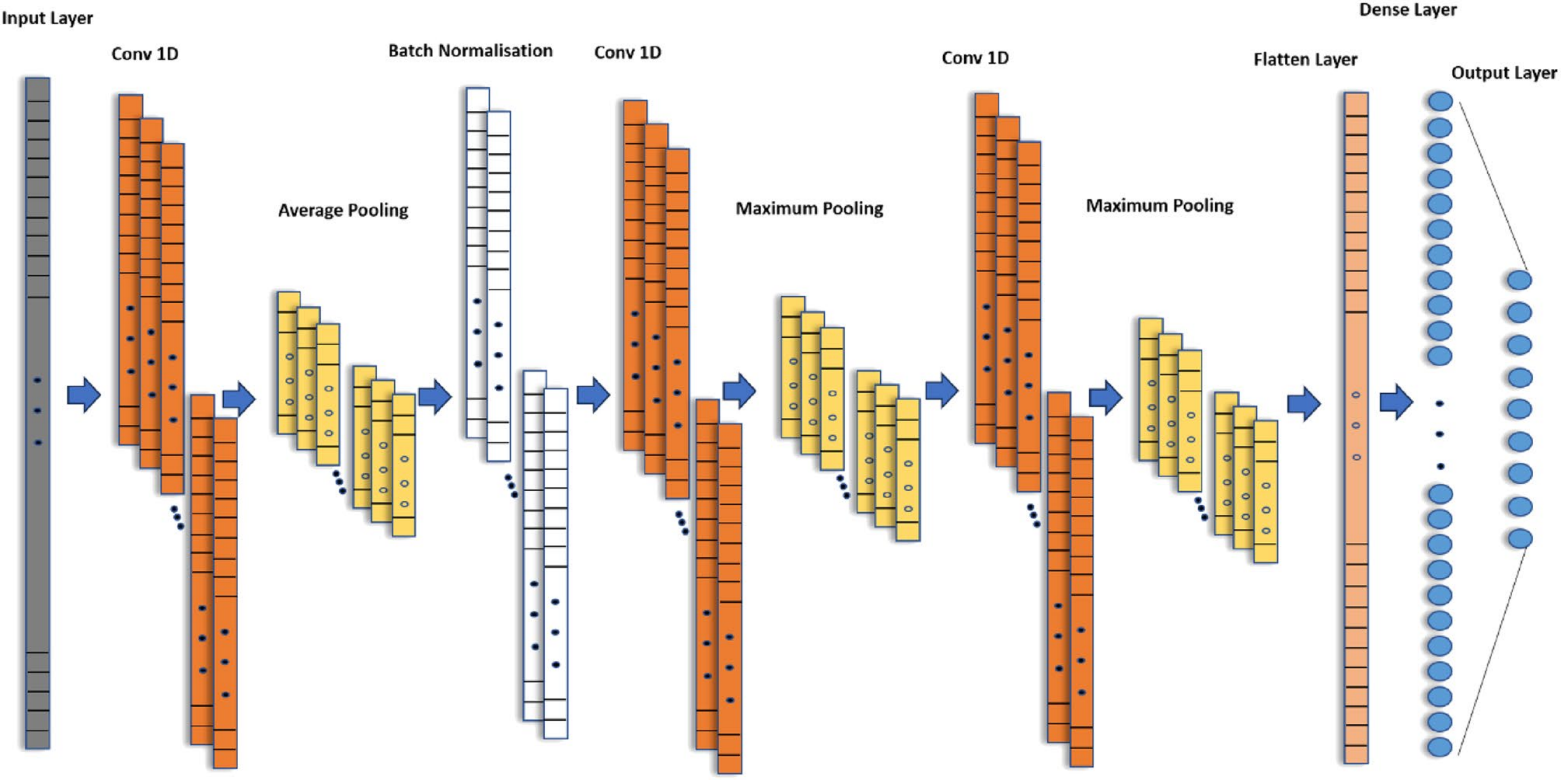
Network structure of DL-MMD.

Exploiting the same concept of filtering, decay rate calculation, signal strength indication through integration, curve analysis, anomaly segregation (not discussed here) and statistical calculation over multiple pulses in time. In summary, the process entails capturing and extracting the local, temporal, and spatial patterns/features collectively. For this AI based algorithms are the most suitable to extract the full potential of information in the received signal. The primary advantage of using this model is transfer learning, which can be integrated with on-spot learning. The latter requires less than a minute to perform soil compensation in a field environment. The intended purpose is to utilize it in future scenarios for real-time learning of unseen classes. Here we will be applying a novel AI based model architecture (DL-MMD) comprising of one-dimensional CNN, as the signal is also one dimensional. The details of the layers within the model can be seen in Table 1 and network structure is illustrated in Fig. 6. The batch size is eight and it refers to the number of samples that are processed together before the model’s parameters are updated. Readers are encouraged to refer^17^ for detailed mathematical insight of CNN for 1-D data.

**Table 1:** Configuration parameters of DL-MMD model.

Property	Setting	Layer config	Dim/size	Param #
Cost func	Categorical cross entropy	Input layer	244 × 1	0
Opt algo	Adamax	Conv1D/Relu, k_1_ = 5, s = 1	244 × 36	216
Mini batch	8	Average-pool 1D, s = 1	240 × 36	0
Regulariser	None	Batch-norm	240 × 36	144
Learn rate (α)	0.001	Conv1D/Relu k_1_ = 5, s = 1	240 × 18	3258
Momentum	0	Max-pooling 1D, s = 1	236 × 18	0
No of examples	10,773	Conv1D/Relu k_2_ = 9, s = 1	236 × 18	2934
Epochs	4000	Max-pooling 1D, s = 9	26 × 18	0
		Flatten	468	0
		FC	32	15,008
		FC/softmax	9	297

Total params: 21,857.

Trainable params: 21,785.

Non-trainable params: 72.

The functionality of each layer has been extensively documented and researched. However, we provide a brief overview of each layer to discuss their intended purpose in the given context. The first layer represents the input to the model which defines the shape and type of the data that will be fed into the model. In our case dataset from nine classes as represented in Eq. (1) for one of the classes is fed into the input layer. It is followed by 1D CNN layer that applies a set of filters to input data, capturing local patterns and extracting relevant features. The kernel size is (k_1_) 5 with stride (s) of 1, activation function used is Rectified Linear Unit (ReLU) and the number of channels is 36. The selection of the number of channels has been thoroughly optimized. Any increase in the number of channels has led to overfitting. It is essential to point out that information in each sample within the received signal curve is not statistically independent from its neighbouring samples as there is a certain amount of correlation due to the process of demagnetizing of sensor coil. For this reason, fully connected layer is not considered at the start as it will lead to overfitting.

AveragePooling1D layer (pool/window size = 5) will perform averaging on the local regions of the output of the previous convolutional layer, allowing the network to capture the average activation within each region. Setting the stride of one will not reduce the dimensionality of data, so maximum information moves to next layer i.e., batch normalization. For the extraction of higher-level features and patterns, again Conv1D layer is used (with reduced 18 channels) and is followed by MaxPooling1D (pool size = 5) with stride of one that selects the maximum value within each pooling window. A third Conv1D layer is added with 18 channels but a different kernel size (k_2_) 9 (actually double of k_1_) with similar stride (s) of 1 is used. It is followed by MaxPooling1D layer with the same pool size and the stride length i.e. 9.

Finally, the flatten layer converts the multi-dimensional representation into a one-dimensional vector, preparing it to be fed into a fully connected (FC) layer i.e. Dense layer. This fully connected layer contains 32 neurons and connects each neuron in previous layer to every neuron within this layer and uses ReLU as activation function. It is then connected to the fully connected (FC) layer at the end, which employs the Softmax activation function and is designed for the classification of inputs into one of nine classes in a supervised manner. By computing probabilities using the provided hot encoded vector labels, it enables the determination of the most likely class for each input using Adamax optimizer. The next section will highlight the result from this learning model architecture.

## Simulations, results & discussions

**Figure 7:**
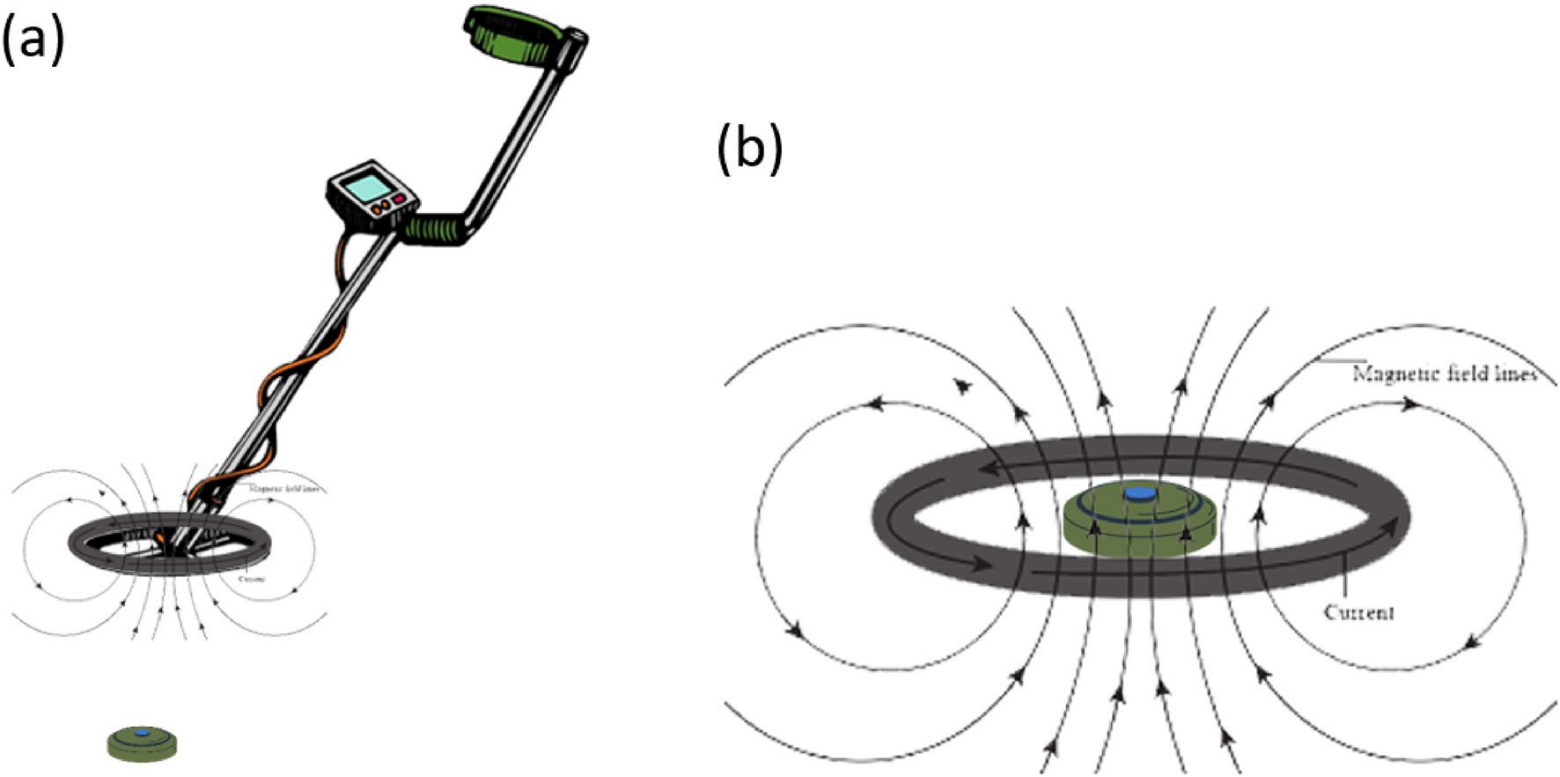
Shows Electromagnetic field simulation of search head in (**a**) and search head in proximity of mine in (**b**).

The experimental arrangement in MMD is a prime factor that defines the integrity of the dataset. The dataset is obtained in lab environment with a PI sensitive coil made up of muti-stranded wire with coil diameter of 170 mm. It is mounted on a transparent acrylic sheet with a miniaturized Tx/Rx (also mounted) at a distance of 100 mm. The electromagnetic field (EMF) simulation of search-head in close proximity of mine is shown in Fig. 7. The received signal is digitized, and synchronized data is obtained for both the transmitted positive and negative pulses. The dataset is then populated with this synchronized pulse data. The pulse repetition frequency, including both pulses, is 880 Hz.The number of pulses M (refer to Eq. (1)) obtained per class is 1330, representing concatenated positive and negative pulses. It is done to simplify the model, with a total number of concatenated samples being N = 244, consisting of 122 samples from each received pulse, respectively. It is approximately 3 s of pulsed data per class.

The samples/targets used to represent the nine classes (previously discussed) include minrl/brick (mineralized soil), sand (non-mineralized soil), APM (standard 0.2 gm) and vertical paper pins (0.2 gm). Mineralization is an indication of magnetic permeability (or susceptibility) of the surface soils that have been exposed to high temperatures and heavy rainfall or water for extended periods of time, often exhibit high mineralization due to the presence of residual iron components. For an in-depth exploration of the magnetic susceptibility across a wide range of soil types, you can find comprehensive information in reference^18^. The choice of using brick, a clay-based material, as a representative sample for mineralized soil is grounded in its unique composition. It contains minerals like iron oxide, such as magnetite or hematite, and exhibits relatively low electrical conductivity^19^. These distinctive characteristics significantly enhance its detectable response when subjected to a MMD. In fact, this response is typically more robust than that of conventional mineralized soil (from which it originates) or even APM. For the sake of simplicity and consistency, we will refer to this material as "minrl" throughout this paper.

**Figure 8:**
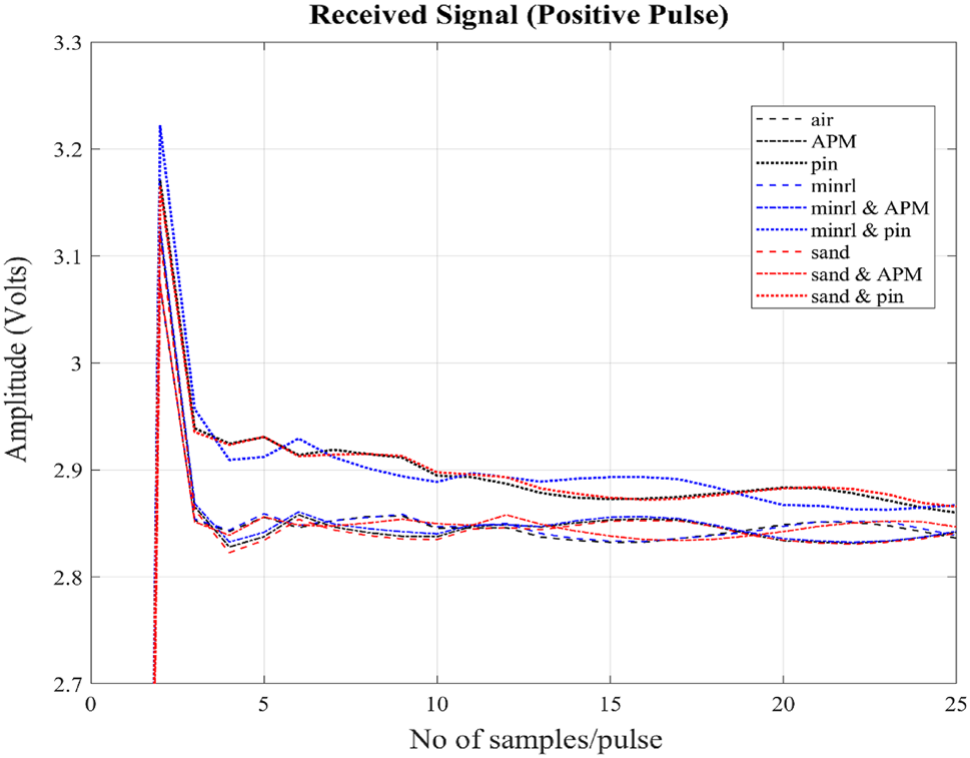
Received signals of a positive transmitted pulse picked up at the sensor coil from the secondary magnetic field produced by the eddy currents induced within the targets. The x-axis shows few numbers of samples (initial part of the signal) per pulse and y-axis shows amplitude of the signal in volts. Signals from nine targets air, APM, pins, minrl, minrl + APM, minrl + pins, sand, sand + APM and sand + pins have been shown.

**Figure 9:**
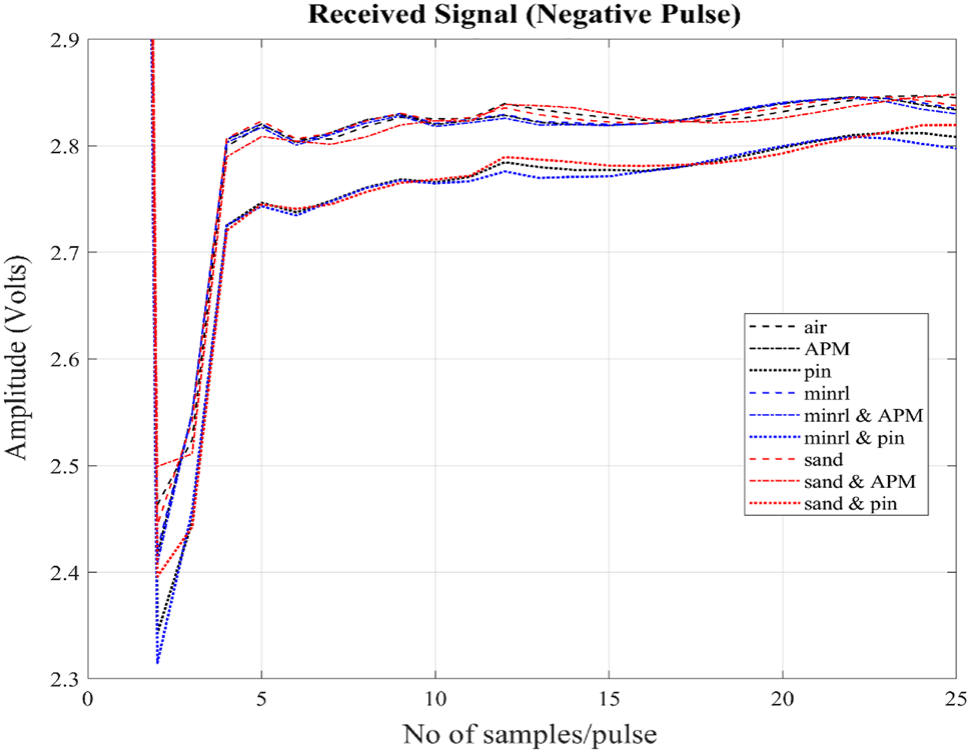
Received signals of a negative transmitted pulse picked up at the sensor coil from the secondary magnetic field produced by the eddy currents induced within the targets. The x-axis shows few numbers of samples (initial part of the signal) per pulse and y-axis shows amplitude of the signal in volts. Signals from nine targets air, APM, pins, minrl, minrl + APM, minrl + pins, sand, sand + APM and sand + pins have been shown.

All of the targets mentioned pose their own challenges, but they are placed in close proximity to the MMD, within a distance of no more than 20 mm parallel to the surface of the coil. The targets are positioned at the center of the coil. The received signals from different target samples of a positive and a negative transmitted pulses can be observed in Figs. 8 and 9 respectively. The figures display a magnified section of the received signal, focusing on the initial samples that are more strongly influenced by the secondary magnetic field compared to later samples. It can also be seen that signals vary in opposite directions as per polarity of the transmitted pulses.

**Figure 10:**
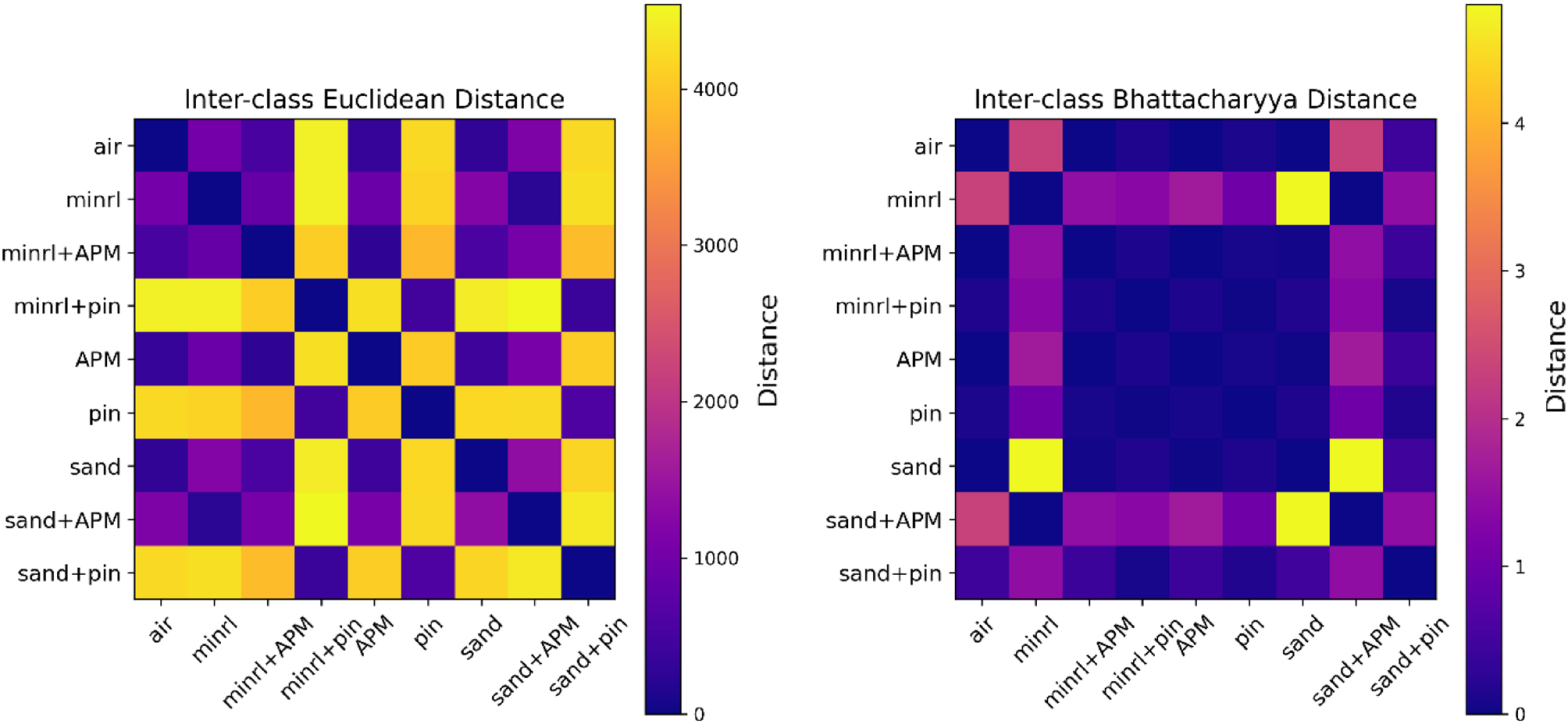
Shows inter-class similarity through Euclidean and Bhattacharyya distances.

The overall dataset comprises a total of 11,970 pulses, representing nine different classes. The dataset is sufficiently diverse, as illustrated in Fig. 10 by examining inter-class distances. For this analysis, two distances are employed: Euclidean distance, which measures point-to-point distance, and Bhattacharyya distance, a metric indicating dissimilarity between two probability distributions. Two cases will be briefly discussed here: one involving the Euclidean distance between air and pins, where the maximum distance is observed as depicted in Fig. 10, which is also evident in the received signal shown in Figs. 8 and 9. The second case pertains to the Bhattacharyya distance between air and sand, illustrating minimal dissimilarity. The impact of this dissimilarity will become evident in the overall results. To prepare this dataset for modelling, these pulses are randomly shuffled and subsequently split into two separate sets: a training dataset containing 10,773 pulses and a validation dataset comprising 1197 pulses.

**Figure 11:**
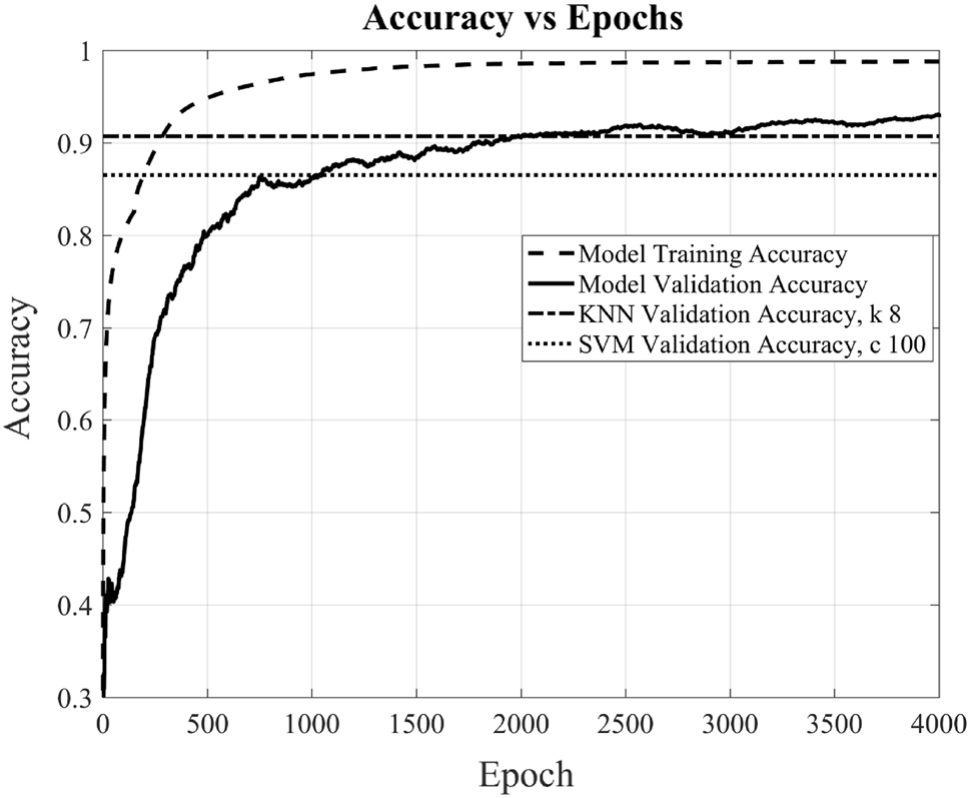
Shows the accuracy and validation accuracy of novel DL-MMD model versus epochs. For comparison, the validation accuracy of KNN and SVM classifier are also shown for k = 8 and C = 100 respectively.

**Figure 12:**
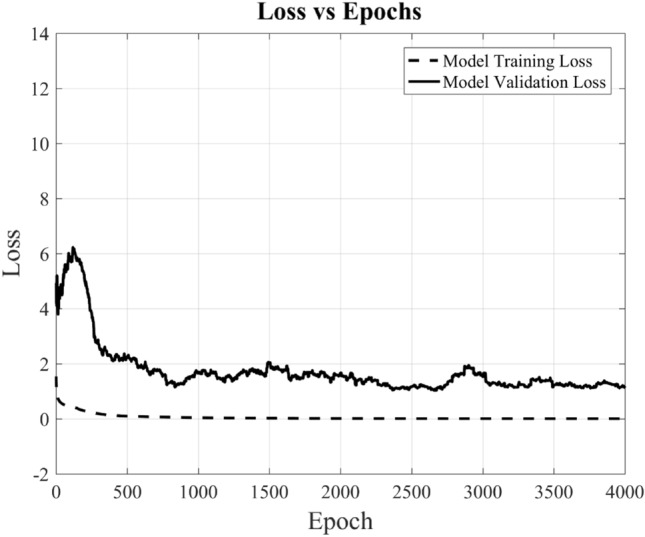
Shows the loss and validation loss of novel DL-MMD model versus epochs.

During the model training phase, input data is structured as a matrix with dimensions [10,773 × 244], and the output, following a supervised learning approach, is provided as a one-hot encoded labeled matrix with dimensions [10,773 × 9]. The accuracy of the trained model on the provided data is tracked across multiple epochs, including both training and validation accuracy. In the context of this training process, one “epoch” signifies a complete iteration over the entire training dataset of size [10,773 × 244], with all training samples processed by the model. Figure 11 depicts the trend, showing that as the training process repeats over multiple epochs, the model steadily enhances its performance and optimizes its parameters. After 4000 epochs, the trained accuracy reaches approximately 98%, while the validation accuracy hovers above 93%. It also shows that the DL-MMD model has more or less converged at 4000 epochs, by achieving the optimum training performance. Likewise, it’s evident that the model’s error loss diminishes with the progression of epochs, as illustrated in Fig. 12.

**Figure 13:**
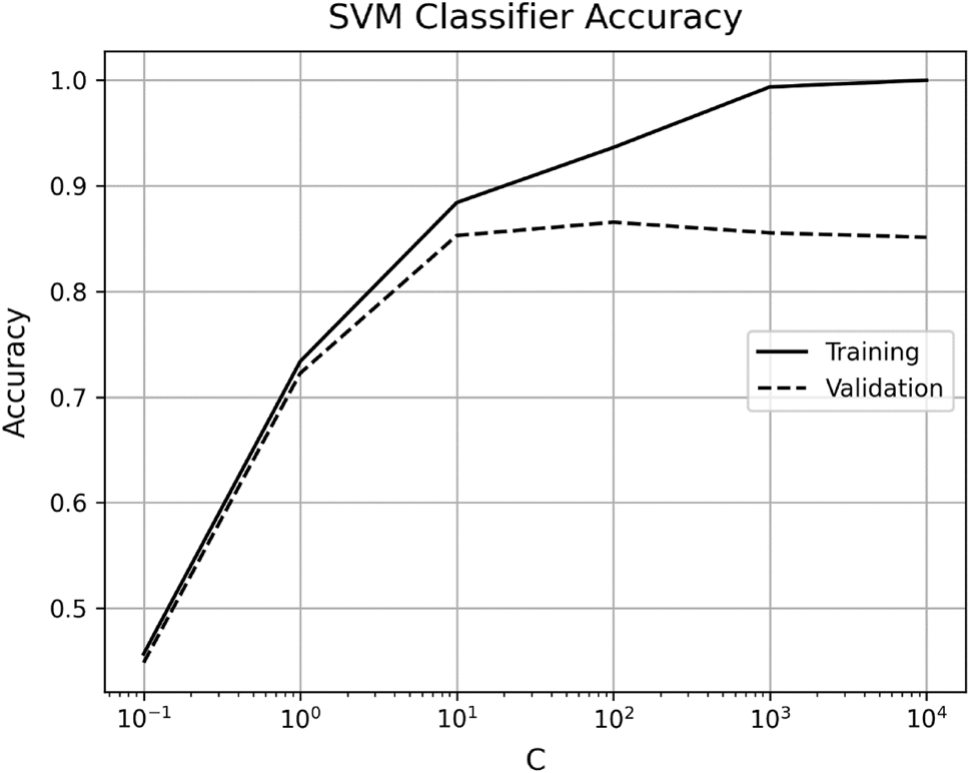
Shows the accuracy of SVM classifier versus regularization parameter C.

Figure 11, also shows that the presented model performs substantially better compared to support vector machine (SVM) and K-Nearest Neighbors (KNN) classifiers. The main working principle of SVM is to separate several classes in the training set with a surface that maximizes the margin (decision boundary) between them. It uses Structural Risk Minimization principle (SRM) that allows the minimization of a bound on the generalization error^20^. SVM model used in this research achieved a training accuracy of 93.6% and a validation accuracy of 86.5%, which is far lower than the performance achieved by the presented model. The parameter for kernel function used is the most popular i.e. radial basis function (RBF) and the value of regularization parameter c optimally selected is 100. The regularization parameter controls the trade-off between classifying the training data correctly and the smoothness of the decision boundary. Figure 13 shows the influence of the regularization parameter c, on the performance of the classifier. The gamma is automatically calculated based on the inverse of the number of features, which ensures that each feature contributes equally to the decision boundary. The hyperparameter optimization is achieved through a manual grid search method. The code iterates through a predefined list of C values [0.1, 1, 10, 100, 1000, 10000], and for each value of C, it trains a Support Vector Machine (SVM) classifier with a radial basis function (RBF) kernel and evaluates its performance on the training and test sets. The accuracy and C values are then plotted to visually check the best performance. It can be seen that the generalization error increases when the value of C is greater than 100, the SVM starts to overfit the training data and thus resulting in decrease in validation accuracy.

**Figure 14:**
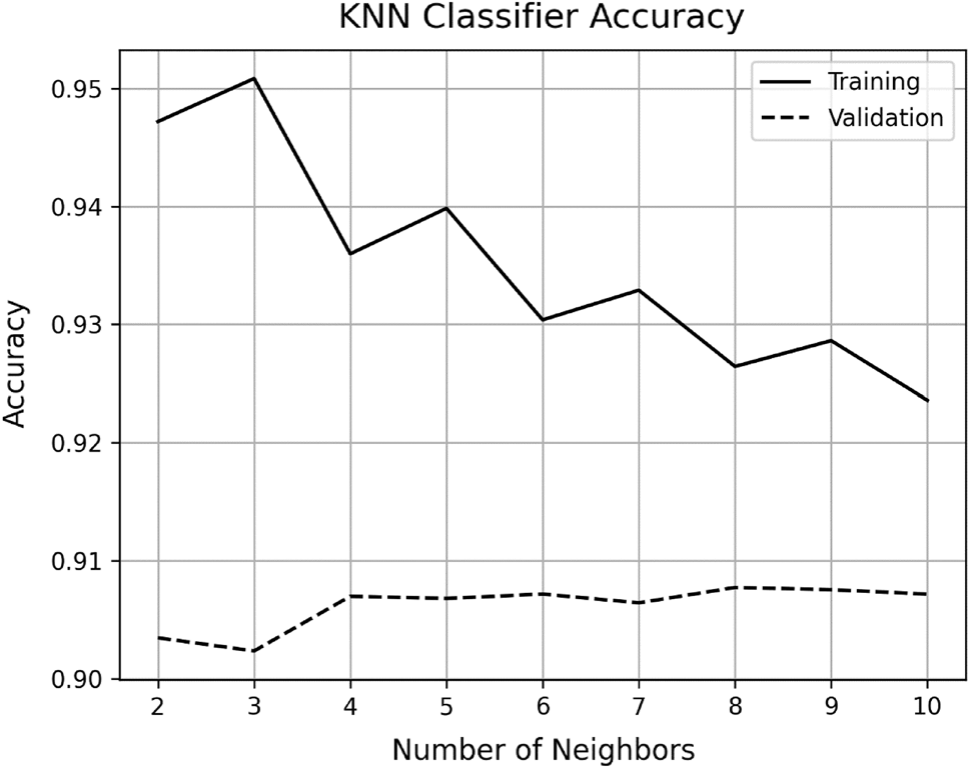
Shows the accuracy of KNN classifier versus number of neighbors k.

While K-Nearest Neighbors (KNN) model with 8 neighbors (k) achieved a training accuracy of 92.6% and a validation accuracy of 90.7% (see Fig. 11), which is lower than the performance achieved by the presented model. To enable comparative analysis, it is essential to showcase the performance of this non-parametric machine learning algorithm. In this context, the algorithm predicts the value of a new data point by considering the majority vote or average of its k nearest neighbors within the feature space^21^. Figure 14 illustrates the influence of the hyperparameter k, the number of neighbors, on the performance of the algorithm. The graph demonstrates that the validation accuracy reaches a maximum of 90.7% when 8 neighbors are considered.

**Figure 15:**
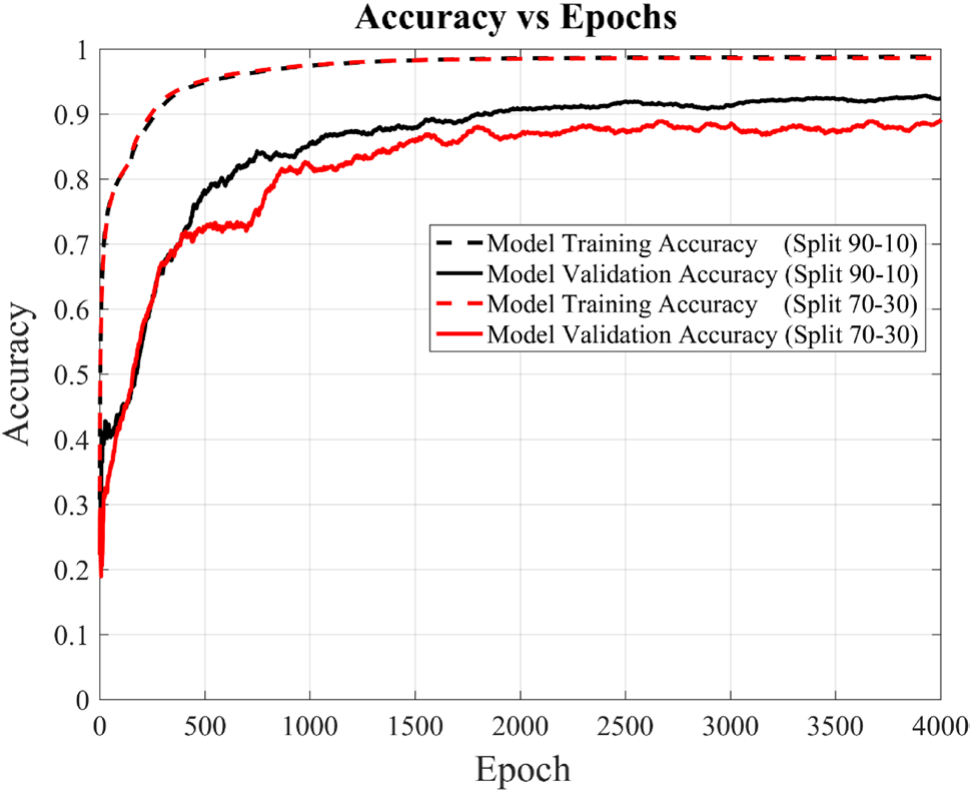
Shows the accuracy and validation accuracy of novel DL-MMD model versus epochs at two different data split ratios i.e. of 90–10 and 70–30.

To further analyze the DL-MMD model versus the experimental data, one more graph has been plotted shown in Fig. 15. This graph illustrates the comparative performance of the presented model using a different data split ratio (70–30), with 70% for training and 30% for validation. The graph shows a slightly degraded performance when compared to the split ratio (90–10) of 90% for training and 10% for validation. However, it still shows validation accuracy of above 88% at 4000 epochs. This degradation is attributed to epistemic uncertainty (model uncertainty) due to slightly less effective learning on a reduced training data and as the training data increases, this uncertainty also reduces.

**Figure 16:**
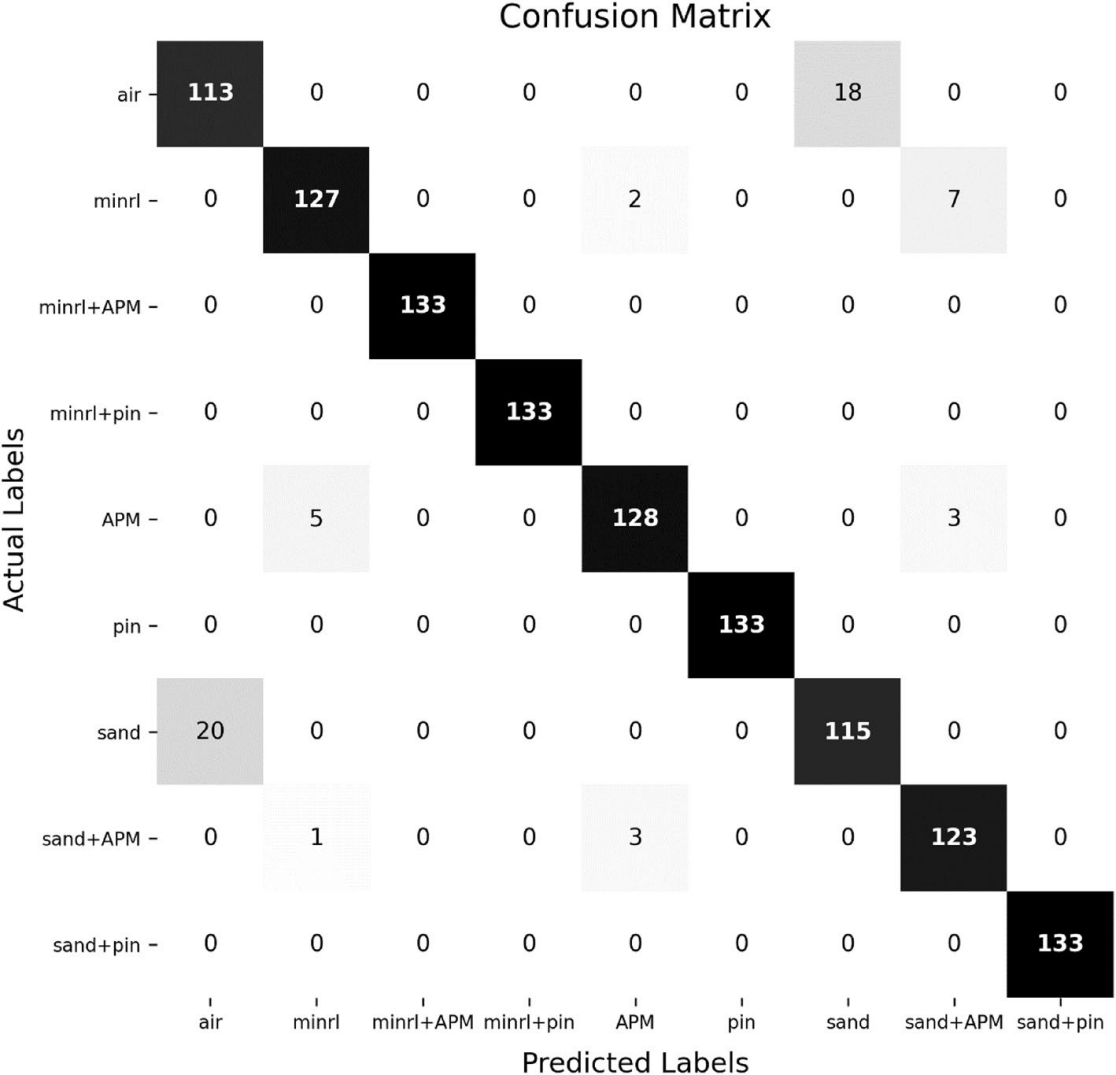
Confusion matrix of the proposed DL-MMD classification on 9 classes.

The performance of the model can also be inferred from the confusion matrix shown in Fig. 16. It provides a tabular representation of the predicted and actual class labels, giving a very important analysis of the models in terms of true positives, true negatives, false positives, and false negatives. For an application perspective of an MMD, safety of the user is of utmost importance for which false negative matters a lot since mine as target must not be missed.. The overall prediction accuracy is above 93.5%, however, for cases of air and sand it is approximately 85 and 86.5% respectively, inferred from the confusion matrix. These two classification cases of relatively less prediction accuracy can be neglected since sand being wrongly classified as air only and vice-versa. These two classes (air & sand) do not trigger any detection alarm by an MMD, thus misclassification of them will not impact efficiency of DL-MMD classifier. It also highlights the fact that sand (of river) has minimal mineralized content and is generally designated as non-mineralised soil. It is therefore difficult to separate the boundary between these two classes in presence of noise and interference.

In addition to this, two further cases need to be examined: one involves mineralized soil (minrl) being wrongly classified as APM, and the other involves APM in sand (sand + APM) being wrongly classified as minrl. The first case is of false positive, it will generate a false alarm and will waste time of the user by requiring unnecessary further investigation. The second case is of more importance i.e. of false negative where an APM is detected but wrongly classified by a DL-MMD and will be discussed in next section. Apart from them, there are minor cases e.g. an APM misclassified as APM in sand (sand + APM), it will not have any impact since target of concern (APM) will remain the same but now being shown buried in sand. The occurrence of all these misclassification cases (apart from the air/sand case & vice-versa) is less than 5% approximately.

**Figure 17:**
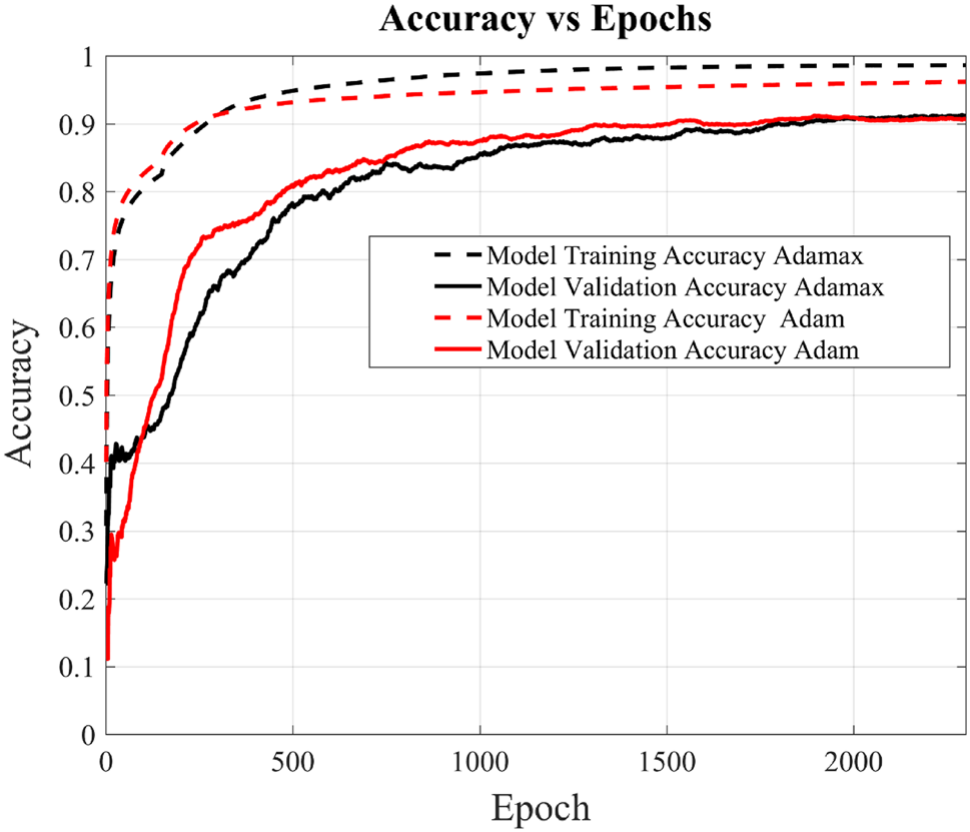
Shows the accuracy and validation accuracy of novel DL-MMD model versus epochs using two different optimizers Adamax and Adam.

These results have been obtained by a substantial dataset based on actual data acquired in two sets of 665 (pulses per class) each obtained at two different times through the experimental setup explained previously and then combined together. Comprehensive simulations have been carried out in the Tensor Flow environment for evaluation of the proposed method. In addition to this, the algorithm has been extensively tested with an increased number of layers and channels, resulting in overfitting. Furthermore, the proposed model has been tested with different optimizers, such as Adagrad, Adamax, and Adam. The comparative analysis of Adam and Adamax can be seen in Fig. 17. Both show equivalent performance after 2000 epochs.

**Figure 18:**
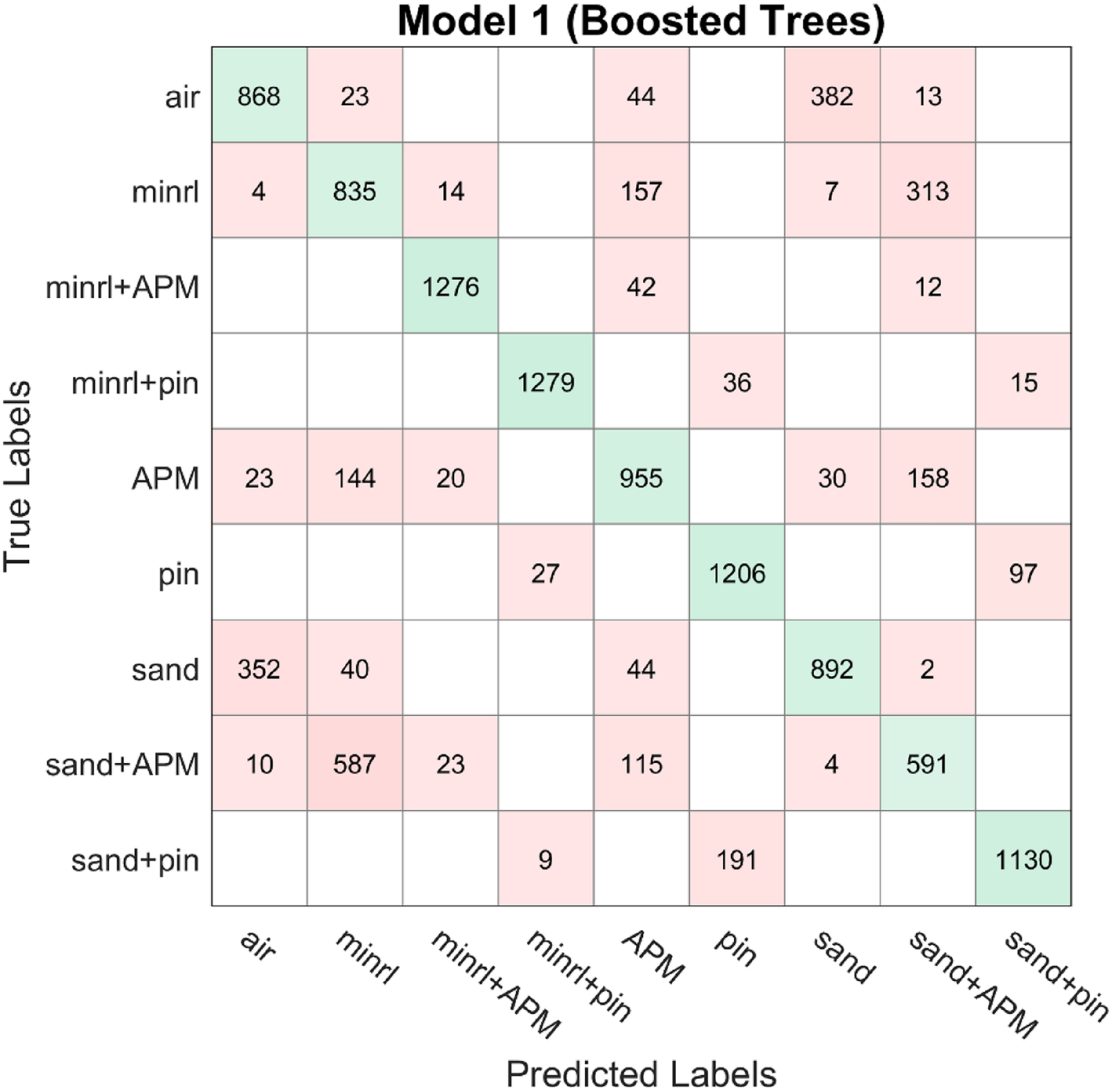
Confusion matrix model 1 AdaBoost.

**Figure 19:**
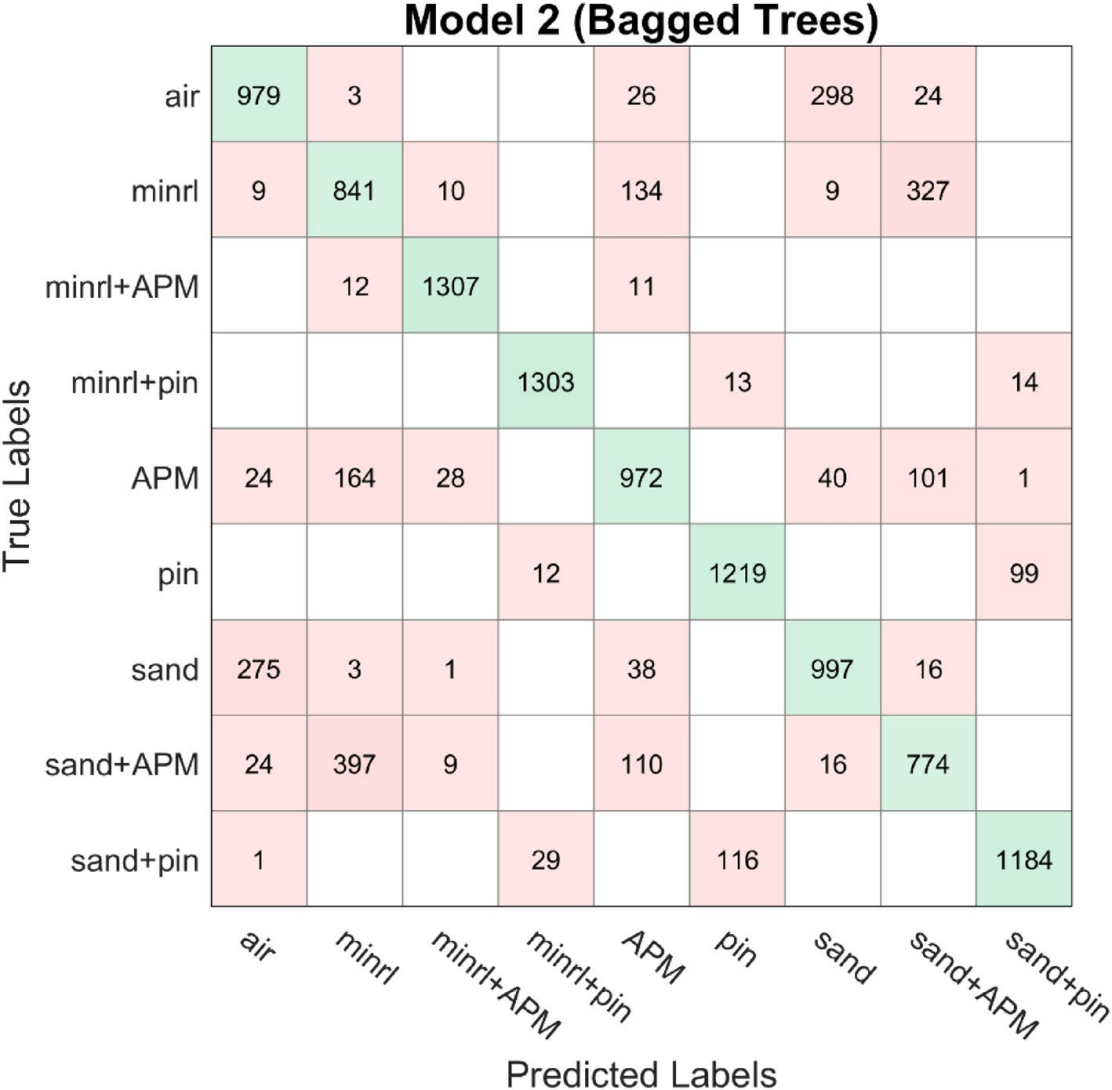
Confusion matrix model 2 Bagged Tree.

**Figure 20:**
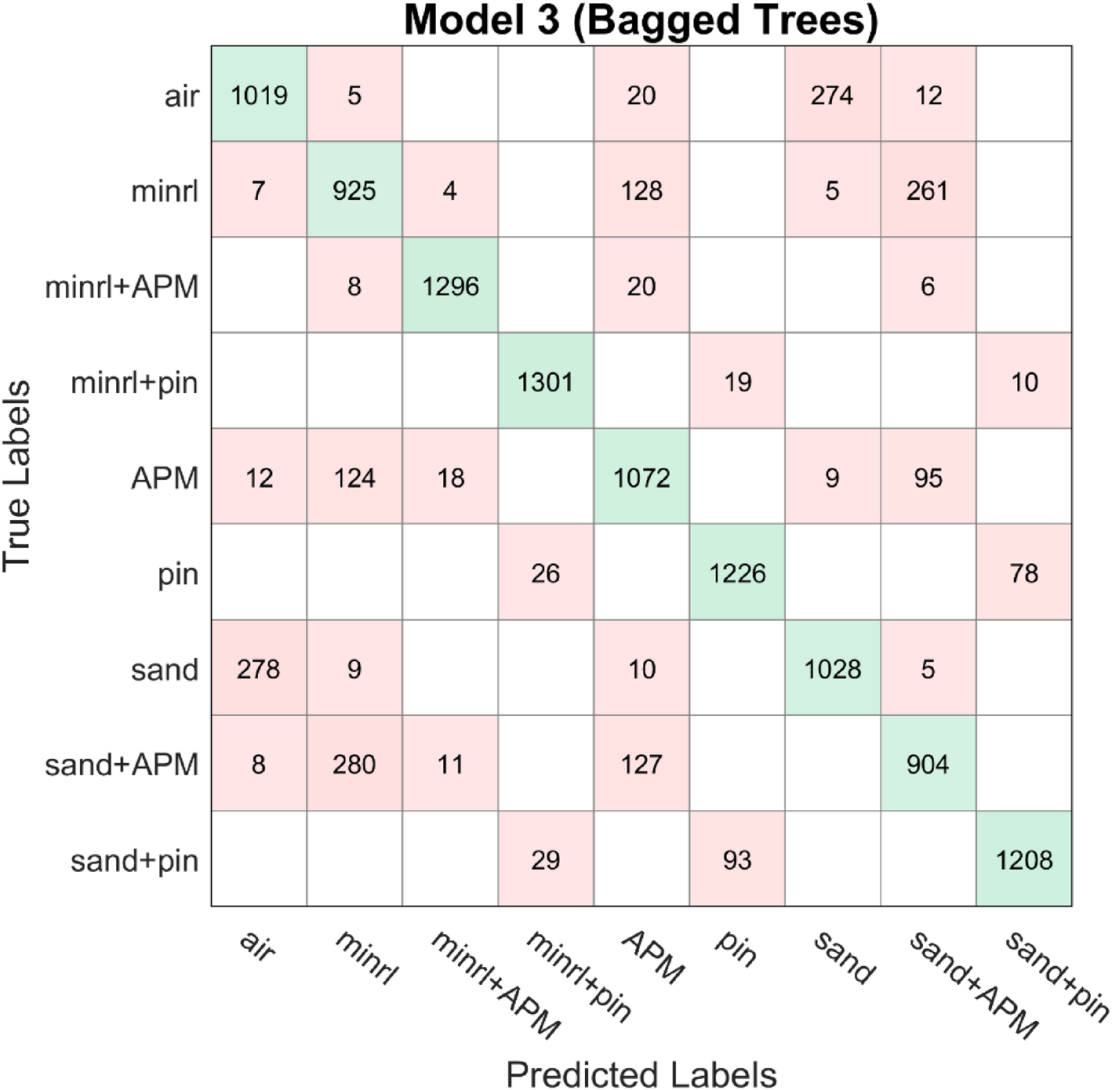
Confusion matrix model 3 Bagged Tree.

In addition to the aforementioned analysis, the dataset underwent evaluation using other prevalent classification algorithms^22^, which utilize the principle of ensemble learning. However, upon comparison, the proposed deep learning architecture exhibited superior performance, achieving an accuracy exceeding 90%. The confusion matrices of these classification algorithms, AdaBoost and Bagged tree, are depicted in Figs. 18, 19, and 20, with the dataset partitioned into an 80/20 ratio, resulting in accuracies of 75.4%, 80%, and 83.3%, respectively. AdaBoost was employed without PCA, utilizing the maximum number of splits and learners set to 30, with a learning rate of 0.1. For Bagged tree, only Model 2 underwent preprocessing with PCA with a variance of 95%. They both utilized the same number of learners as AdaBoost and a maximum split of 11,969.

It is pertinent to mention that there is always redundant information within the received signal that creates background bias, especially in sensitive areas with low metal content. Information regarding the detection of APM mines buried at different depths is available (in the parameter decay rate), but it is not utilized. Therefore, for an APM buried at a different depth (relative to the search head) to the one it is trained on, there is a chance that it can be misclassified. The information exists, but it needs to be pre-processed before feeding the signal to the model. One approach could be to use focused AI models, similar to those shown in Ref^23^, that inject synthetic bias into the signal to generalize the model in our case at different depths. Another approach can be to localize the area with different decay rates, similar to the one shown in Ref^24^ for 2D image application. One of the future work will be to utilize this information and integrate it into the DL_MMD architecture.

## Conclusion

In this paper, we present a new approach using deep learning for distinguishing between anti-personnel mines (APM) and small vertical paper pins (0.2 g) in both mineralized soil and non-mineralized environments like air or sand. Our method is particularly important for practical demining operations, aiming to minimize both false positives and false negatives. This ensures more accurate target classification, ultimately expanding the search area.

Our innovative learning framework combines the pulse induction method with Convolutional Neural Networks (CNN) to act as a classifier for post-detection targets, providing a unique advantage. It has shown success in classifying mineralized soil and has the potential for extension to other soil types. The system demonstrates an impressive validation accuracy of 93.5% across nine distinct classes.

However, a challenge remains in accurately classifying APM, especially in scenarios like an APM buried in sand (sand + APM), where there is a 5.2% false negative rate, often misclassified as mineralized soil (minrl). To address this, we suggest that the operator’s decision should not solely rely on the deep learning (DL) model’s classification, given its post-detection nature. Instead, the operator’s experience using the Mine Metal Detector (MMD) should be considered. Despite this challenge, the DL model boasts a high confidence rate of 94.8% in correctly classifying the target (sand + APM), providing valuable information to enhance operational efficiency. For fully automated mine detection, a multisensory approach can ensure complete reliability in classifying mines based on metal content and material density.

To further enhance our model’s accuracy and expand its classification capabilities, future work will involve incorporating a broader range of mine types and increasing the magnetic footprint through spatial measurement diversity by introducing another target position, such as the side of the coil. This expansion will double the dataset with minimal modification to the DL-MMD algorithm. An advantage of using DL-MMD is that standard mine characteristics, such as metal content, size, shape, and orientation, remain consistent regardless of burial location. This consistency significantly boosts the algorithm’s accuracy and reliability in real-world scenarios.

## Data Availability

The datasets generated and/or analysed during the current study are available in the repository, https://github.com/MinhasSF/MMD_AI/.
